# Exploring the Efficacy of Alirocumab and Evolocumab in Reducing Low-Density Lipoprotein (LDL) Cholesterol Levels in Patients With Familial Hypercholesterolemia: A Systematic Review

**DOI:** 10.7759/cureus.28930

**Published:** 2022-09-08

**Authors:** Gaurav Luthra, Mahrukh Shahbaz, Halah Almatooq, Paul Foucambert, Faith Esbrand, Sana Zafar, Venkatesh Panthangi, Adrienne R Cyril Kurupp, Anjumol Raju, Safeera Khan

**Affiliations:** 1 Internal Medicine, California Institute of Behavioral Neurosciences & Psychology, Fairfield, USA; 2 Dermatology, California Institute of Behavioral Neurosciences & Psychology, Fairfield, USA; 3 Pediatrics, California Institute of Behavioral Neurosciences & Psychology, Fairfield, USA

**Keywords:** pcsk-9 inhibitor, ldl cholesterol, evolocumab, alirocumab, familial hypercholesterolemia

## Abstract

Patients with familial hypercholesterolemia (FH) have an increased risk of having abnormally high low-density lipoprotein cholesterol (LDL-C) levels. One of the main groups of drugs used for FH is statins. However, even with statins, most patients with FH do not achieve their pre-defined therapeutic LDL-C goals. Therefore, proprotein convertase subtilisin/kexin type 9 inhibitors (PCSK9i) serve to decrease LDL-C levels in that population.

A total of 838 articles were found after searching the databases of PubMed, MEDLINE, and Cochrane Library. After including only full-text peer-reviewed articles published in the last 10 years, 67 articles remained. Thirteen articles were put through the Cochrane bias assessment tool to screen for bias. After a strict quality assessment based on the criteria, eight articles were extracted and included in this systematic review.

The data extraction from these studies showed that alirocumab and evolocumab were efficacious in decreasing LDL-C levels and achieving the pre-defined LDL-C goals. Many parameters influenced the strength of the LDL-C reduction: sample size of the population, genetic structure of the patients affected by FH, length of the trial, or baseline lipid-lowering therapy used. Therefore, one must consider several other factors while evaluating the percent reduction of PCSK9i. This review is limited because it did not comment on these drugs' cardiovascular outcomes or mortality benefits. In addition, some of the articles used in this systematic review have small sample sizes and short trial times, limiting the long-term evaluation of these drugs.

## Introduction and background

Familial hypercholesterolemia (FH) is a common inherited metabolic disorder because of genetic mutations, which eventually cause drastically elevated low-density lipoprotein cholesterol (LDL-C) levels [1]. FH can be categorized into heterozygous and homozygous forms, and both of them present with their unique set of symptoms and treatments [2]. Heterozygous FH (HeFH) is found in approximately one in 500 individuals [1]. On the other hand, homozygous FH (HoFH) is rarer and found in approximately one in 1,000,000 [1]. 

It is crucial that patients are properly screened and early diagnosis followed by strict medical intervention and lifestyle changes because those left untreated are at greater risk of premature myocardial infarction (MI) and coronary heart disease (CHD) [3]. Diagnosis of HeFH is based upon several criteria: family history, presence of any CHD in the patient's history, presence of xanthomas and corneal arcus, abnormally elevated levels of LDL-C seen on several measurements, and/or the presence of the genetic mutation detected [4]. However, physical exam findings such as xanthomas and corneal arcus are not always found in the patients. Still, the presence of such signs in a young patient (<45 years) should cause the clinician to look further into the diagnosis of FH [1].

In addition to the clinical diagnosis of FH, there are three well-known formal criteria for making the diagnosis. These include the Simon Broome criteria, the United States "Make Early Diagnosis to Prevent Early Deaths" (US MEDPED) diagnostic criteria, and the Dutch Lipid Clinic Network diagnostic criteria [4]. The main things that Simon Broome criteria consider are cholesterol and low-density lipoprotein (LDL) concentrations, physical exam characteristics such as tendon xanthomas, molecular diagnosis, and family history of MI or cholesterol levels. However, a disadvantage of these criteria is that they fail to detect HeFH due to mutations in apolipoprotein B-100 (Apo B-100) and proprotein convertase subtilisin/kexin type 9 (PCSK9) [5]. The Dutch Lipid Clinic Network criteria are developed to deal with the shortcoming of the Simon Broome criteria and consider the molecular defect of FH and the points of the Simon Broome criteria [5]. The US MEDPED criteria only consider the patient's age and specific endpoints of total cholesterol levels and do not consider the mutation analysis or the physical signs like xanthomas [5]. More details on the specific points considered in each of the criteria can be found in the review done by Al-Radadi et al. [5].

Upon diagnosis, treatment should begin as soon as possible to lower the LDL-C levels by at least 50% [2]. Since one of the genetic mutations leading to FH is a loss-of-function mutation in LDL receptors, 3-hydroxy-3-methylglutaryl-coenzyme A (HMG-CoA) reductase inhibitors (statins) are one of the main drugs used to lower LDL cholesterol in patients with FH [6]. HMG-CoA reductase is a rate-limiting enzyme in the synthesis of cholesterol in the liver; therefore, blocking it causes the sensor molecule, sterol regulatory-element binding protein 2 (SREBP2), to increase the expression of LDL receptors on the surface and, as a result, decreases the LDL concentration in the blood [6,7]. Theoretically, the cells will continue to take up LDL-C if statins are taken every day; however, this will disturb the cholesterol homeostasis in the cells [7]. To prevent this situation, SREBP2 causes the release of PCSK9, which causes the degradation of LDL receptors (whose expression is increased due to statin use) [6,7]. PCSK9 inhibition will increase the recycling of LDL receptors and hence LDL uptake, proving an effective strategy to lower LDL cholesterol.

In addition, it is recommended that patients with HeFH who are at high cardiovascular risk should aim to maintain their LDL-C levels at <70 or <100 mg/dL [8]. However, studies have found that despite using statins and other lipid-lowering therapies such as ezetimibe and/or bile acid sequestrants, only approximately 20% of the patients with HeFH achieve LDL-C levels of <100 mg/dL [9,10]. Therefore, PCSK9i lowers the LDL-C levels of such patients into the therapeutic range.

## Review

Methods

The methods and results for this systematic review are reported based on the Preferred Reporting Items for Systematic Reviews and Meta-Analysis (PRISMA) guidelines following screening selection.

Search Strategy

We used the electronic databases PubMed, MEDLINE, and Cochrane Library to look for articles using Medical Subject Headings (MeSH) and keywords to obtain the most relevant reviews and studies for analysis. The keywords included: "PCSK9 Inhibitors", "Alirocumab," "Evolocumab," "Familial Hypercholesterolemia," or "Hyperlipoproteinemia Type ll." We used several combinations of Boolean to put together the keywords according to the algorithm used in PubMed. The MeSH search used to obtain the articles was: (("Proprotein Convertase 9"[Majr]) OR "Proprotein Convertases/antagonists and inhibitors"[Majr]) AND ( "Hyperlipoproteinemia Type II/drug therapy"[Majr] OR "Hyperlipoproteinemia Type II/prevention and control"[Majr] OR "Hyperlipoproteinemia Type II/therapy"[Majr] ). The articles were then screened to highlight those most relevant to the search question and selected according to the inclusion/exclusion criteria.

Inclusion and Exclusion Criteria

**Figure 1:**
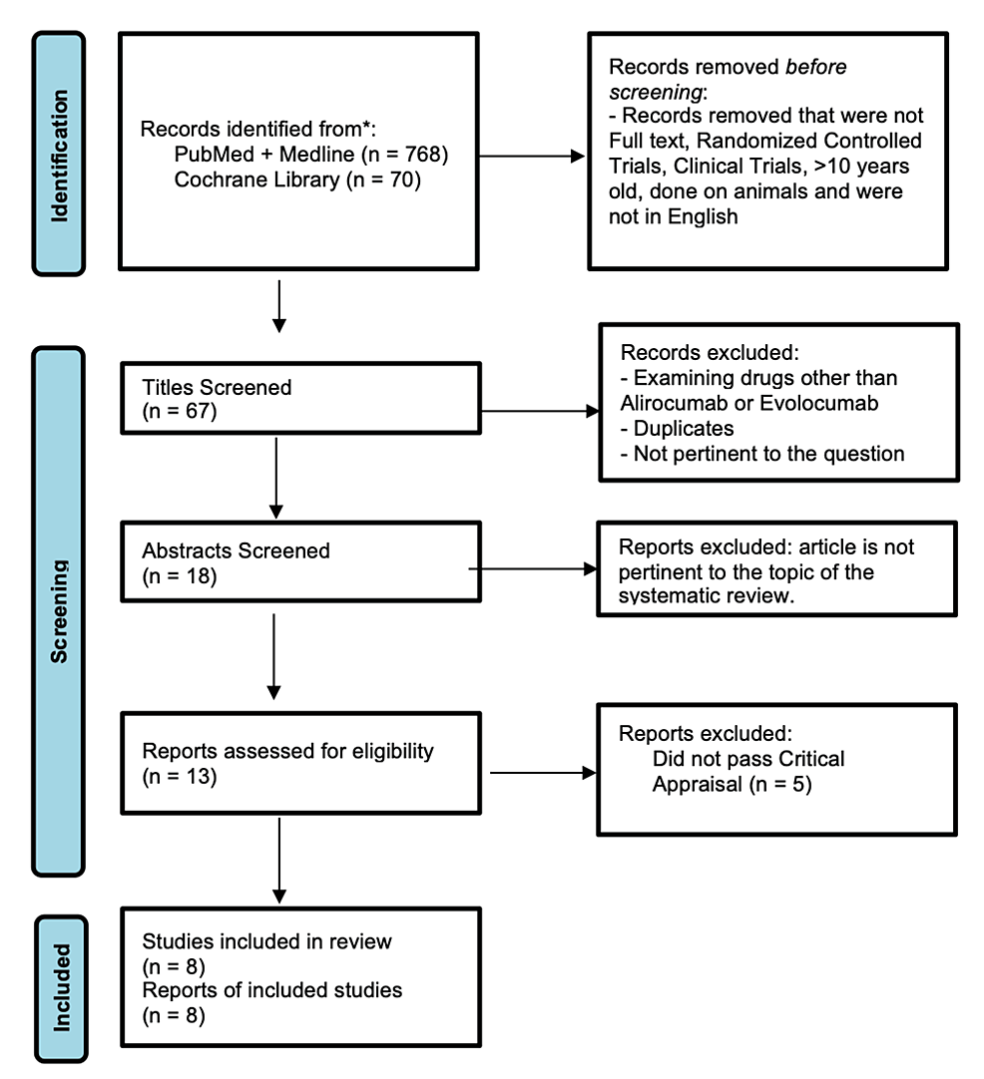
Preferred Reporting Items for Systematic Reviews and Meta-Analyses (PRISMA) flow diagram

The articles analyzed in this systematic review used the following inclusion criteria: (1) papers from the past 10 years; (2) randomized controlled trials (RCTs) and clinical trials; (3) published in English; (4) human subjects; (5) full reports (not just abstracts). To exclude papers, the following criteria were used: (1) unpublished literature; (2) grey literature; (3) papers discussing pharmacokinetics or pharmacodynamics of the PCSK9 inhibitors; or (4) papers discussing any drugs except evolocumab or alirocumab. The flow chart showing the methodology used to select articles for inclusion is explained in Figure 1.

Article Screening

Initial screening was done by going through the title or abstract to weed out the articles deemed unqualified. After that, the inclusion and exclusion criteria described above were used. The remaining articles were read in detail, checked using the Cochrane risk assessment, and finalized to be included in the review.

Critical Appraisal of Studies

We critically appraised our screened articles using the Cochrane risk-of-bias tool. The bias risk assessment looked at seven causes of potential bias, and a summary for each clinical trial in this review is given in Table 1.

**Table 1:** A summary of risk-of-bias assessment for the studies in this review using the Cochrane assessment tool

Study	Bias from the randomization process	Bias in the recruitment of participants	Bias from the effect of assignment to intervention	Bias from the effect of adhering to an intervention	Bias due to missing outcome data	Bias in measurement of outcome	Bias in reported results	Overall risk
Blom et al., 2020 [11]	Low Risk	Low Risk	Low Risk	Low Risk	Low Risk	Low Risk	Low Risk	Low Risk
Ginsberg et al., 2016 [12]	Low Risk	Low Risk	Low Risk	Low Risk	Low Risk	Unknown	High Risk	Low Risk
Hovingh et al., 2017 [13]	Low Risk	High Risk	Low Risk	Low Risk	Low Risk	Low Risk	Low Risk	Low Risk
Kastelein et al., 2015 [14]	Low Risk	Low Risk	Low Risk	Low Risk	High Risk	Low Risk	Low Risk	Low Risk
Kastelein et al., 2017 [15]	Low Risk	Low Risk	Low Risk	Low Risk	Low Risk	Unknown	Low Risk	Low Risk
Raal et al., 2014a [16]	Low Risk	Low Risk	Low Risk	High Risk	Low Risk	Low Risk	High Risk	Low Risk
Raal et al., 2014b [17]	Low Risk	Low Risk	Low Risk	Low Risk	Low Risk	Low Risk	Low Risk	Low Risk
Santos et al., 2020 [18]	Low Risk	Low Risk	Unknown	Low Risk	Low Risk	Low Risk	Low Risk	Low Risk

Results

A total of 838 articles were generated from keywords, MeSH, eligibility criteria, and databases. Of the 838 articles, 768 were from PubMed, and 70 were from Cochrane Library. After applying the basic inclusion criteria, only 67 articles remained on PubMed. PubMed and Cochrane library articles were then screened by their titles and abstracts. Fifty-four articles were screened out because of topic irrelevance and duplicates. Thirteen articles were critically appraised using the Cochrane bias assessment tool. Five were removed because they did not meet 70% of the assessment criteria. Eight articles met the criteria and they were only RCTs. The PRISMA flow diagram is shown in Figure 1 [19].

The reviewed clinical trials differed in design, population, and primary endpoints. However, the efficacy in lowering to a target LDL level and adverse events associated with the PCSK9i was a common part of each RCT. This is summarized in Table 2.

**Table 2:** An outline summary of the randomized clinical trials. LDL-C: low-density lipoprotein cholesterol; Apo B: apolipoprotein B-100; Lp(a): total cholesterol lipoprotein(a); HDL-C: high-density lipoprotein cholesterol; ApoA1: apolipoprotein A1; CV: cardiovascular; HeFH: heterozygous familial hypercholesterolemia; HoFH: homozygous familial hypercholesterolemia; MI: myocardial infarction; CRP: C-reactive protein

Study	Inclusion Criteria	Intervention	Primary Outcome	Secondary Outcome	Conclusions
Blom et al., 2020 [11]	Clinical or genetic diagnosis of HoFH and LDL-C >70mg/dl	69 patients were randomized 2:1 to alirocumab or placebo	Percent reduction from baseline in LDL-C vs. placebo after 12 weeks of treatment	Percent change from baseline in Apo B, non-HDL-C, total cholesterol Lp(a), HDL-C, triglycerides, and ApoA1 at week 12.	Significant and clinically meaningful reductions in LDL-C in patients suffering from HoFH.
Ginsberg et al., 2016 [12]	Patients diagnosed with HeFH and LDL-C levels greater than 160mg/dl. In addition, they must also be taking a maximum dose of statin for at least four weeks.	Subcutaneous alirocumab 150mg vs. placebo every two weeks for 78 weeks.	Percent reduction in LDL-C in comparison to the baseline at week 24.	Percent change from baseline in non-HDL-C, total cholesterol Lp(a), HDL-C, triglycerides.	The intervention demonstrated significant reductions in LDL-C for patients suffering from HeFH who are taking the maximum tolerated dose of stain and other lipid-lowering therapies.
Hovingh et al., 2017 [13]	LDL-C >/=2.6 mmol/L despite statin therapy with or without ezetimibe.	Patients from the RUTHERFORD trial Re-randomized 2:1 to get evolocumab in addition to standard of care vs. standard of care for 52 weeks.	Percent changes in LDL-C from baseline to 40 weeks.	N/A	Long-term usage of evolocumab is efficacious and well-tolerated and successfully sustains long-term LDL-C reduction.
Kastelein et al., 2015 [14]	Patients diagnosed with HeFH through genotyping or clinical criteria and no history of CV events or those with MI or ischemic stroke in the past. Everyone received high-dose statin therapy with or without other medications to lower lipid levels for at least four weeks.	Randomized 2:1 to 75 mg alirocumab in comparison to placebo every two weeks. Stratification was done by the history of MI or ischemic stroke, statin treatment, and geographic region.	Percent change in LDL-C from baseline to week 24.	Change in Apo B, non-HDL-C, Lp(a), triglycerides, HDL-C, and ApoA1.	The treatment caused a significant reduction in LDL-C from baseline by week 24 compared to placebo. The treatment was generally well-tolerated throughout the treatment (78 weeks).
Kastelein et al., 2017 [15]	Included patients with HeFH from the following RCTs: FH l (NCT01623115), FH ll (NCT01709500), LONG TERM (NCT01507831), and HIGH FH (NCT01617655). Patients diagnosed with HeFH are on a maximally-tolerated statin, and other lipid-lowering therapies from the above four 78-week ODYSSEY trials are analyzed.	FH l&ll: Placebo vs. 75mg Alirocumab every two weeks (with a dose increase to 150 mg every two weeks at week 12 if week 9 LDL >/=70 mg/dL).	Compared to the baseline, the percentage change in LDL-C at week 12, week 24, and week 78.	Effect of treatment on other lipid parameters: non-HDL-C, Apo B, triglyceride, total cholesterol, Lp(a), HDL-C, and ApoA1. Number of patients with high cardiovascular risk achieving a set LDL-C goal of <70mg/dL and <100mg/dL at weeks 12, 24, 52, and 78.	Treatment with alirocumab caused significant reductions in LDL-C for patients with HeFH receiving statin with or without other lipid-lowering therapies.
Raal et al., 2014a [16]	Male or female patients 12 years or older were diagnosed with HoFH (genetic or clinical criteria).	Randomized 2:1 to either subcutaneous injection of 420 mg evolocumab vs. placebo every four weeks.	Percent change in LDL-C at week 12 in comparison to the baseline.	Percent change compared to the baseline at week 12 for ApoB, HDL-C, and triglycerides.	Patients with HoFH receiving the intervention significantly reduced LDL-C by 31% and Apo B by 23% compared to placebo.
Raal et al., 2014b [17]	Three hundred thirty-one patients between the ages of 18-80 years who had HeFH (clinical criteria) were also taking lipid-lowering therapy for at least four weeks with LDL-C levels of 2.6 mmol/L.	Subcutaneous 140 mg evolocumab every two weeks or 420 mg evolocumab monthly vs. placebo every two weeks or months for 12 weeks.	Percent changes in LDL-C at week 12 compared to baseline and the mean LDL-C changes at weeks 10 and 12.	Percentage of patients attaining LDL-C goal of less than 1.8 mmol/L. Mean percent change compared to the baseline for apolipoproteins, other lipids, and high sensitivity CRP.	There was an LDL-C reduction of 60% compared to the placebo, and greater than 60% of the patients reached the target LDL goal of less than 1.8 mmol/L.
Santos et al., 2020 [18]	Patients between the age of 10-17 years were diagnosed with HeFH and had previously received lipid-lowering therapies for at least four weeks. These patients must also have an LDL-C level of 3.4 mmol/L or more and triglycerides of 4.5 mmol/L or less.	A subcutaneous monthly injection of 420mg evolocumab vs. placebo for 24 weeks.	Percent change in LDL-C at week 24 in comparison to the baseline.	Mean percent change in LDL-C at weeks 22 and 24 compared to the baseline. Absolute change in LDL-C at week 24 in comparison to the baseline. Percent change in non-HDL-C, Apo B, total cholesterol to HDL-C ratio, and Apo B to ApoA1 at week 24 compared to baseline.	There was a significant reduction in LDL-C in patients diagnosed with FH and treated with evolocumab.

Our systematic review included only RCTs and clinical trials. The eight RCTs chosen for review were randomized, double-blind, placebo-controlled trials. The articles' primary outcome was the percent changes in LDL-C concentration at several different set periods. However, the secondary endpoints included a mixture of percent changes: apolipoprotein (Apo) B, non-high-density lipoprotein cholesterol (non-HDL-C), total cholesterol lipoprotein(a) (Lp(a)), high-density lipoprotein cholesterol (HDL-C), triglycerides, or apolipoprotein A (ApoA1). In addition, all the articles also commented on the safety of the alirocumab or evolocumab administration by mentioning the number and type of adverse events encountered. All of the articles commented on the safety of the drugs by examining the injection-site reactions and treatment-emergent serious adverse events.

Efficacy

Blom et al. selected 59 (85.5%) patients taking high-intensity statin, 50 (72.5%) patients on ezetimibe, 10 (14.5%) on lomitapide, and 10 (14.5%) on apheresis [11]. Regarding the risk factors, 30 (43.5%) patients had a history of coronary artery disease (CAD) [11]. It is also important to know the method used to diagnose the patients with the type of FH; 42 (60.9%) patients were diagnosed with HoFH genetically, and 27 (39.1%) patients were diagnosed clinically (in-study genotyping), and 17 (24.6%) patients could not be diagnosed with HoFH [11]. On average, patients in the treatment group had an LDL-C level of 295.0 mg/dL compared to 259.6 mg/dL in the placebo group [11]. The reduction in the LDL-C in the treatment group in comparison to placebo at week 12 is statistically significant, measured as the least squares mean difference of -35.6% +/- 7.8% (p < 0.0001) [11]. There was also a significant difference in the least-squares mean reductions in the treatment group vs. placebo at week 12 in the level of Apo B (29.8%), non-HDL-C (32.9%), total cholesterol (26.5%), and Lp(a) (28.4%) [11].

Ginsberg et al. too selected all patients receiving statin therapy (72.9% were on a high-intensity statin) [12]. There were 24.3% patients who were receiving ezetimibe and 107 patients were randomized to the treatment vs. placebo group [12]. At week 24, there was a statistically significant LDL-C least-squares mean difference of -39.1% (p < 0.0001) between the treatment group and placebo [12]. There was also significant difference in least-squares mean reductions in the treatment group vs. placebo at week 24 in the level non-HDL-C (35.7%, p < 0.0001), total cholesterol (28.4%, p < 0.0001), Apo B (30.3%, p < 0.0001), and Lp(a) (14.8%, p = 0.0164) [12].

Hovingh et al. selected 440 patients to receive the evolocumab treatment vs. placebo (standard of care (SOC)) [13]. Of the patients, 28% had CAD, and 13.9% had cerebrovascular or peripheral arterial disease; 79% of the patients received high-intensity statin therapy, and 64.3% received ezetimibe in addition to statin therapy [13]. Because it was an open-label extension of previously done RCTs, it did not include the statistical analysis but rather the absolute reduction in LDL-C and other lipid parameters. Patients in the treatment group (evolocumab plus SOC) had a mean 53.6% reduction in LDL-C after 48 weeks compared to a mean increase of 2% in the placebo [13]. Reductions in Apo B, non-HDL-C, triglycerides, and Lp(a) were also observed in the treatment, along with an increase in HDL-C [13].

Kastelein et al. selected 735 patients and randomized them into two studies, FH l and FHII with 486 patients and 249 patients,respectively [14]. Forty-six percent of the patients in FH l and 36% in FH ll reported having CHD. More than 80% of patients were also taking a high-dose statin, and more than half were also receiving ezetimibe 10 mg in addition to the high-dose statin. In FH l, there was a statistically significant LDL-C reduction of 57.9% (p < 0.0001) in comparison to the baseline. In FH ll, there was a statistically significant LDL-C reduction of 51.4% (p < 0.0001) in comparison to the baseline [14]. 

In 2017, Kastelein et al. included patients who had been enrolled in four 78-week ODYSSEY studies: FH l (NCT01623115), FH ll (NCT01709500), LONG TERM (NCT01507831), and HIGH FH (NCT01617655) [15]. A total of 1257 patients were randomized to receive either alirocumab or placebo. The number of patients with a history of diabetes, hypertension, and premature CAD was higher in the LONG TERM and HIGH FH trials than in the FH l and FH ll [15]. In addition, baseline LDL-C, Apo B, total cholesterol, and non-HDL-C were higher in the LONG TERM and HIGH FH trials compared to FH l and FH ll [15]. At week 24, the average change in LDL-C concentration from baseline was -48.8% in the patients treated with alirocumab 75/150 mg and -55% for patients treated with alirocumab 150 mg every two weeks (p < 0.0001 for both) [15]. In addition, about 75.3% of the patients treated with 75/150 mg every two weeks and 64.5% of the patients treated with 150 mg every two weeks achieved the LDL-C goal of <70 or <100 mg/dL (p < 0.0001) [15].

Raal et al. included 50 patients in their study, who were randomized to receive evolocumab (n=33) or placebo [16]. Genotyping was done on all patients, and everyone who had met the clinical diagnosis was confirmed genetically for HoFH except for one patient, who turned out to be heterozygote genetically. Everyone was receiving statin treatment at baseline, with 46 patients taking a high-intensity statin and 45 taking ezetimibe [16].

In the same year, 2014, Raal et al. included 331 patients in another study, who were randomly assigned to receive evolocumab 140 mg once every two weeks, evolocumab 420 mg monthly, or placebo once every two weeks. Of the enrolled patients, 42 were women and 89% were white [17]. Around 31% of the patients had CAD, 87% were taking high-intensity statin doses, and 62% were also taking ezetimibe. Administration of 140 mm of evolocumab resulted in 59.2% of LDL-C mean reductions at week 12 compared to the baseline. Monthly administration resulted in a mean LDL-C reduction of 61.3%. At week 12, 68% of the patients in the 140 mg evolocumab group reached LDL-C lower than 2 mmol/L, and 63% of the patients in the evolocumab monthly group.

Santos and co-authors had 158 randomized patients, and 157 patients in their clinical trial, who either received evolocumab (104 patients) or a placebo (53 patients) [18]. Eighteen patients had greater than two risk factors contributing to atherosclerotic cardiovascular disease, 52 had a first-degree family member with a history of atherosclerotic cardiovascular disease, 104 patients received a genetic diagnosis of FH. Twenty-four patients took high-intensity or moderate-intensity statin, and 21 took ezetimibe. At week 24, there was a -44.5% mean percent change in LDL-C with the treatment group and -6% with the placebo group, with a difference of -38.3%. The treatment group also showed benefits in terms of non-HDL-C and Apo B.

Discussion

This review examined the efficacy of PCSK 9 inhibitors, alirocumab and evolocumab, in patients with FH (heterozygous or homozygous). Statins have been the drug of choice for HeFH for decades; however, there are still patients who either cannot tolerate statins or are just not adequate to lower LDL-C levels to the therapeutic range. For instance, in a cross-sectional study conducted in the Netherlands on 1249 patients with HeFH, approximately 96% were taking statin drugs; however, only about 26% reached the LDL-C goal of <2.5mmol/l [20]. These findings are a precise indicator that there is a need for an adjunct treatment to help patients with HeFH to bring their LDL-C levels to the therapeutic range. PCSK9 inhibitors fill this gap and can be used as an adjunct with statin drugs to treat patients with HeFH.

Efficacy

This systematic review of PCSK9 inhibitor drugs (alirocumab or evolocumab) found statistically and clinically significant reductions of different magnitudes in LDL-C when added to statin therapy in patients diagnosed with FH. Even though all of the studies used in the systematic review showed some form of LDL-C reduction in the treatment arm compared to the placebo, the strength of this result is based on the characteristic of the population tested and the drug used. Out of the eight articles we analyzed in this systematic review, five had patients diagnosed with HeFH, and three used patients diagnosed with HoFM. In patients with HeFH, the trials for alirocumab had a larger number of patients than the trials evaluating evolocumab (1257 vs. 440). In addition, studies evaluating alirocumab also ran for a longer time than evolocumab (78 weeks vs. 52 weeks or 12 weeks). The lower number of patients can introduce higher variability in results, which might not be seen in a study with a higher number of patients. The studies analyzing patients with HeFH showed that the PCSK9 inhibitors reduced LDL-C significantly and brought patients' LDL-C to a pre-defined goal. In addition, it significantly decreased the levels of triglycerides, total cholesterol, non-HDL-C, Apo B, and Lp(a) and increased the levels of HDL-C, which are the healthy lipoproteins. It is well known that high levels of LDL, the bad cholesterol, are a major cause of heart disease; therefore, these drugs help patients reduce the risk for cardiovascular disease. We will first analyze the articles about HeFH and then move on to HoFH.

In 2016, Ginsberg et al. conducted a trial to evaluate the efficacy of alirocumab in a specific group of patients who, in particular, had a high level of LDL-C at screening. They explored the difference between their results and other clinical trials with similar experiments to explain that the strength of LDL-C reductionneeds to be evaluated in the context of the population examined and the design of the experiment. They noticed that the LDL-C reductions were lower than in the ODYSSEY program, including those with FH patients [12]. Even though all of the trials we examined mentioned reduction in LDL-C, the reduction's strength is different based on factors such as lipid-lowering therapies that the patient is on, the baseline LDL-C to start with, and several others. In his article, Ginsberg and colleagues noticed that three of the study sites (with 20 randomized patients) closed down due to breaches in Good Clinical Practice; and when the data from these sites were omitted from the analyses, a greater reduction in LDL-C was noticed (-49.8% vs. -39.1%) [12]. Ginsberg et al. randomized only 107 patients for only 24 weeks; therefore, even though there was a statistically significant reduction, the smaller number of patients can introduce variability in the results, and it is difficult to measure the long-term effects with a length of only 24 weeks [12]. Therefore, we must look at the efficacy of alirocumab in patients with HeFH according to trials that had a greater number of participants and ran for a longer term.

In 2015, Kastelein et al. had randomized 735 patients in two studies: one with alirocumab 75 mg and another with alirocumab 150 mg [14]. The studies also ran longer than that of Ginsberg et al.(78 weeks vs. 24 weeks). In 2017, Kastelein et al. had the single largest collection of patients (1257) with HeFH studied in the phase-3 clinical trial [15]. In comparison to the analysis done by Ginsberg et al. [12], both of these studies had a greater LDL-C reduction (Kastelein et al. (2015) with 57.9% reduction in FH l and 51.4% in FH ll at week 24 and Kastelein et al. (2017) with 48.8% for patients on alirocumab 75/150 mg and 55% for those taking alirocumab 150 mg) [14,15]. According to Faber et al., if the sample size of a paper is too small, it may prevent the findings in the paper from being applied to a larger population outside the study population [21]. Because the purpose of these clinical trials is to use the data from the studies to treat patients with FH, small sample sizes in studies such as Ginsberg et al. may make it difficult. However, the reduction seen in the articles discussed above is still clinically and statistically significant, and the results must be evaluated with the population and drug characteristics in mind.

Hovingh et al. and Raal et al. discussed the efficacy and safety of evolocumab in patients diagnosed with HeFH [13,17]. Compared to the trials on alirocumab in patients with HeFH, the two trials for evolocumab used in the systematic review had fewer patients (440 and 415). They were conducted for a shorter duration (52 weeks and 12 weeks) [13,17]. This can have implications in extrapolating the data; however, the reductions were still significant in bringing the LDL-C to its goal. Hovingh et al. evaluated two different dosages of evolocumab (140 mg once in two weeks or 420 mg monthly) and showed that both the doses achieved similar reductions in LDL-C (~60%) [13]. Another aspect is that all the articles mentioned in their methods are the ways (clinically or genetically) and the proportion of patients diagnosed with either HeFH or HoFH. It seems obvious the need to classify patients based on their diagnosis type; however, Raal et al. suggest that genetic analysis might not help assess the response to evolocumab in patients with HeFH as it is in HoFH [17]. In their study, about 20% of patients were detected to have no mutations about HeFH and 3% of the patients with mutations in both LDL receptor alleles, signifying HoFH. However, they were clinically diagnosed with HeFH. Despite this non-mutation and discrepancy between genetic and clinical diagnoses, these patients responded equally well to the evolocumab treatment as those with evidence of defective mutations. However, we will examine whether the response of LDL-C reduction in patients with HoFH correlates with LDL receptor genotype. This finding is important because it allows them to conclude that genetic analysis might not be significant when assessing the evolocumab response in patients with HeFH [17]. Therefore, in addition to examining the dosing of the drug, sample size, and characteristics of the patient population, it might also be important in the future to account for the discrepancy between genetic diagnosis and clinical diagnosis of the patients to evaluate the effectiveness of the treatment in patients with FH to reproduce the findings suggested by Hovingh and colleagues [13].

This systematic review has two papers on HoFH: one examining the efficacy and safety of alirocumab [11], and one examining the efficacy of evolocumab [16]. Compared to the articles discussing HeFH, these articles for HoFH analyze a smaller population; 69 patients in the research by Blom et al. [11] and 50 patients in the study by Raal et al. [16]. In addition, both these studies only ran for 12 weeks. The results of these studies must be evaluated with this information in context. A smaller population can make it difficult to extrapolate the data, and a shorter period can make it difficult to conclude the long-term effect of the treatment. However, Raal and colleagues did address this as a study limitation [16]. They mentioned that the long-term safety of evolocumab in HoFH would be addressed in an open-label long-term extension where a larger cohort would be selected. Both studies show a significant reduction in LDL-C with the treatment compared to the placebo. As mentioned earlier, it is helpful to obtain genetic confirmations in patients with HoFH and can be helpful for select patients. Raal and colleagues showed a higher reduction in LDL-C in patients who were recorded to have two LDL receptor defects rather than just a single LDL receptor-negative mutation [16].

On the other hand, unclassified patients in terms of genetic mutation had a variable LDL-C reduction (ranging from -56.6% to 42.9%) [16], suggesting that there must be different types of mutations in those patients. Therefore, in patients with HoFH, it is valuable to confirm the exact type of mutations present genetically. On the other hand, Blom and colleagues reported a significant reduction in LDL-C at week 12 with the use of alirocumab in comparison to the baseline (35.6%); however, the reduction observed was small in comparison to the other studies done, for example, 61% in the ODYSSEY long-term trial [11]. However, the difference was that the ODYSSEY trials were done for patients with HeFH and non-familial hypercholesterolemia; therefore, the reduction can be attributed to the genetic deficiencies in patients with HoFH. In addition, the response seen with alirocumab was variable in terms of the LDL-C reductions [11]. Therefore, compared to evolocumab, the treatment of patients with HoFH with alirocumab has a significant reduction, although not as robust. Although the reductions observed with alirocumab are helpful, greater reductions are usually needed, especially in patients with HoFH.

Santos and colleagues showed a significant reduction in the LDL-C in their trial of pediatric patients diagnosed with HeFH and treated with evolocumab in addition to statin therapy [18]. Like the other studies for the HoFH, the number of patients randomized for this trial was small and were evaluated for a short term; hence, the drug's long-term effect must be analyzed in a separate study.

Limitations

As can be seen from each of the studies used in this systematic review, the patients were on some lipid-lowering therapy at baseline (high-intensity statin, moderate-intensity statin, or ezetimibe) in addition to the PCSK9 inhibitor used in the trial. The patients were taking varying doses of different strength statins, and PCSK9 inhibitors were used because these patients were not reaching the target LDL-C levels. However, in reality, there is a step-wise approach implemented, where the current therapy the patient receives is maximized, and if it is not tolerated, another drug is added. Some of the studies analyzed did not use this approach, so it would be valuable for future studies to follow this protocol. In addition, this systematic review did not analyze the relation of the PCSK9 inhibitors to reducing cardiovascular events or mortality. Lastly, despite the benefit of using a RCT, some of the reviews used in this systematic review continued the experiment using an open-label treatment design. Open-label treatment designs can introduce bias into the experiment that cannot be eliminated because of the lack of comparative control.

## Conclusions

PCSK9 inhibitors (alirocumab and evolocumab) have proven themselves to be efficacious drugs. A significant portion of the patients reached their pre-defined LDL-C goals, and several other lipid parameters were significantly improved. Even though all trials showed a reduction in LDL-C with alirocumab or evolocumab, the strength of this reduction depends on the sample size and length of the trial. Some of the articles in this analysis have a small sample size making it difficult to extrapolate the data. Short trial lengths also make it difficult to comment on the long-term efficacy of the drugs. In addition, most patients were on baseline statin therapy (high-intensity statin, moderate-intensity statin) or ezetimibe, and a step-wise approach to using PCKS9 inhibitors (maximizing the dosage of the current lipid-lowering therapy before starting PCSK9 inhibitors) would be beneficial. However, high cardiovascular-risk patients might not have enough time to make a step-wise approach. In addition, long-term drug safety and mortality benefits of the PCSK9 inhibitors should be considered in future studies. In conclusion, these PCSK9 inhibitors are efficacious and potent drugs for FH.
